# The Views of Clients and Healthcare Professionals on the Perceived Effectiveness of HIV Prevention Programmes in Selected O. R. Tambo District Municipality Clinics

**DOI:** 10.3390/idr18050114

**Published:** 2026-10-07

**Authors:** Thina Sinalo Mntonintshi, Asanda Boboyi

**Affiliations:** 1 School of Public Health, Faculty of Medicine and Health Sciences, iYunivesithi Walter Sisulu, Mthatha 5099, South Africa; 215190904@mywsu.ac.za; 2 Department of Social Work, Faculty of Law, Humanities and Social Sciences, iYunivesithi Walter Sisulu, Mthatha 5099, South Africa

**Keywords:** clients, health belief model, health care professionals, HIV prevention programmes, pre-exposure prophylaxis, Sustainable Development Goals 3

## Abstract

Aim/Background: This study examined the perceived effectiveness of HIV prevention programmes in the O.R. Tambo District Municipality from the perspectives of both clients and healthcare professionals. Although a range of interventions is available—such as condom distribution, HIV testing, pre-exposure prophylaxis (PrEP), and prevention of mother-to-child transmission (PMTCT)—HIV prevalence in the area remains high. Methods: A qualitative, exploratory design guided by the Health Belief Model (HBM) was employed to explore the factors influencing the utilisation of these services. Data were collected through semi-structured interviews with clients and focus group discussions with healthcare professionals. Participants were selected using purposive sampling, and the data were analysed thematically. Results: The findings show that the perceived effectiveness of these programmes is shaped by several factors, including adherence, service accessibility, stigma, and levels of knowledge and awareness. While participants generally acknowledged the benefits of the programmes, their impact was limited by inconsistent use, restricted access, and prevailing misconceptions. Conclusions: The study concludes that there is a need to strengthen educational efforts, improve access to services, and address social barriers in order to enhance programme utilisation and ultimately reduce HIV transmission.

## 1. Introduction

Human immunodeficiency virus (HIV) is a virus that weakens the immune system by attacking CD4 cells, which play a key role in the body’s defence against infections [1,2]. It is mainly transmitted through unprotected sexual intercourse, the sharing of contaminated sharp objects such as needles or syringes, and from mother to child during pregnancy, childbirth, or breastfeeding [3]. Although there is currently no cure for HIV, it can be effectively managed with antiretroviral therapy (ART), which reduces the viral load and improves the quality of life of those living with the virus [4].

Globally, an estimated 39.9 million people were living with HIV at the end of 2023, with approximately 0.6% of adults aged 15–49 years affected [5]. The World Health Organization African Region (WHO AFRO) remains the most affected, with around 3.4% of adults living with HIV and accounting for more than two-thirds of all people living with HIV worldwide [3]. Eswatini has the highest national HIV prevalence, with about 25.9% of adults living with the virus [6]. South Africa, however, has the largest number of people living with HIV, estimated at 7.8 million in 2022, representing about 13% of the population, although its prevalence rate is lower than that of Eswatini [7].

Within South Africa, the O.R. Tambo District Municipality in the Eastern Cape is characterised by high levels of poverty and HIV prevalence. The district has a poverty rate of approximately 80.4% and an HIV prevalence of 11.4% as of 2022 [8]. It is largely rural, with limited healthcare infrastructure and restricted access to essential services. In some communities within the district, HIV prevalence among adults exceeds 20%. This is linked to ongoing socio-economic challenges, limited access to healthcare, and persistent stigma related to HIV testing and treatment [9].

HIV continues to disproportionately affect disadvantaged populations. The stigma associated with the disease often leads to social isolation, discourages individuals from seeking healthcare, and limits access to primary healthcare services [3]. These factors have wider socio-economic consequences. HIV can reduce productivity and slow economic development, particularly when affected individuals are unable to work due to illness. In many cases, households experience income loss, especially when the infected individual is the main source of financial support. At the same time, the increased demand for healthcare services places additional strain on already constrained health systems [6].

Despite the introduction of various HIV prevention programmes—such as health education, condom distribution, HIV testing services, pre-exposure prophylaxis (PrEP), and post-exposure prophylaxis (PEP)—new infections remain a major public health concern [10,11,12]. These interventions have been integrated into primary healthcare systems to improve access, reduce stigma, and provide more comprehensive care [6]. However, there is still a need to understand how these programmes function in practice. Therefore, this study aims to explore the perspectives of clients and healthcare professionals to assess the perceived effectiveness of these interventions.

### 1.1. Problem Statement

Despite the availability of HIV prevention services such as pre-exposure prophylaxis (PrEP), condom use, HIV testing, prevention of mother-to-child transmission (PMTCT), and voluntary medical male circumcision, as well as global initiatives like the Sustainable Development Goals (SDG 3), important challenges still persist. SDG 3 aims to ensure healthy lives and promote well-being for all, with a specific target of ending the AIDS epidemic by 2030 through improved prevention, treatment, and strengthened healthcare systems [13].

However, HIV continues to be a major public health concern, with prevalence rates remaining high in many settings despite these ongoing efforts. This situation suggests that existing programmes may not be as effective as intended or are not being fully utilised by the populations they are meant to serve. As a result, there is still a significant gap in understanding why HIV rates remain elevated despite the wide implementation of prevention strategies.

In response to this gap, this study seeks to explore the perceived effectiveness of HIV prevention programmes from the perspectives of both healthcare professionals and clients. Gaining insight into how clients perceive and engage with these services is important for improving their utilisation, enhancing client satisfaction, and identifying barriers to participation. Such understanding can contribute to the development of more responsive and effective HIV prevention interventions.

### 1.2. Justification of the Study

This study is significant as it seeks to explore the experiences of individuals who regularly test for HIV, as well as the perceptions of healthcare professionals who provide these services, which is important for improving HIV prevention and service delivery.

- This study will benefit clients in the rural communities by helping identify barriers that hinder perceived effectiveness and access to these services, so that better interventions are developed that will actually meet the needs of the population. The findings of this study will provide insights into the challenges HCPs face when administering these services, while promoting more responsive, client-centred care that enables better communication and trust between clients and HCPs.
- The evidence of this research can inform policy-making processes by shaping and refining public health interventions. Client feedback on the perceived effectiveness of HIV prevention programmes is essential, as it highlights successes and gaps in service delivery and informs improvements in implementation.

### 1.3. Theoretical Framework

This study is guided by the Health Belief Model (HBM), a widely recognised psychological framework used to explain and predict health-related behaviours, particularly the uptake of preventive health services [14,15]. The HBM provides a useful framework for understanding why individuals may choose to engage with, or avoid, HIV prevention programmes.

According to the model, health-related behaviours are influenced by individuals’ perceptions of their susceptibility to a condition, the severity of that condition, the benefits of taking preventive action, and the barriers that may hinder such action [14,16]. In the context of HIV prevention, these elements are highly relevant. Decisions to use prevention methods such as condoms, HIV testing, or PrEP are shaped by how individuals perceive their risk of infection, the seriousness of HIV, and the perceived advantages and challenges associated with these interventions.

Applying the HBM in this study provides a structured way of interpreting participants’ views on HIV prevention services. It allows for a deeper understanding of how individuals perceive their risk of HIV and how these perceptions influence their engagement with available services. For instance, individuals who consider themselves at low risk may be less likely to use preventive services, whereas those who perceive higher risk and severity may be more motivated to take preventive action.

The model also helps to explain how perceived barriers—such as stigma, fear of side effects, or limited access to services—can discourage participation in HIV prevention programmes, even when these services are available. At the same time, factors such as increased awareness, education, and recognition of the benefits of prevention strategies can encourage greater engagement. As noted by [17], the HBM remains a valuable tool in public health research for designing and evaluating interventions to promote positive health behaviours.

In this study, the HBM was used as an interpretive lens during data analysis. The identified themes were examined in relation to HBM constructs, including perceived benefits, perceived barriers, cues to action, and self-efficacy, to provide a deeper understanding of the factors influencing the uptake and perceived effectiveness of HIV prevention programmes among clients and healthcare professionals.

### 1.4. Aim and Objectives

The study explores and describes the views of clients and health care professionals on the perceived effectiveness of HIV prevention programmes in selected clinics within the O.R. Tambo District Municipality. The following objectives will guide the study:

- To explore and describe the views of clients and health care professionals on the perceived effectiveness of HIV prevention programmes.
- To provide a way forward and recommendations to strengthen the perceived effectiveness of HIV prevention programmes

## 2. Materials and Methods

### 2.1. Approach

This study adopted a qualitative research methodology, which is appropriate for exploring and understanding human experiences, social contexts, and complex phenomena [18]. Qualitative research focuses on collecting non-numerical data through methods such as interviews, focus group discussions, and observations. This approach enables researchers to gain a deeper and more detailed understanding of participants’ perspectives [18,19].

A qualitative approach is suitable for this study, as it examines both clients’ views and healthcare professionals’ perspectives on HIV prevention programmes. It allows for in-depth exploration and flexibility, enabling the capture of participants’ experiences and insights in a meaningful way. This is particularly important for understanding how individuals perceive and engage with these programmes within their specific social and contextual settings [20].

The reporting of this qualitative study was guided by the Consolidated Criteria for Reporting Qualitative Research (COREQ), and the completed COREQ checklist is provided as Supplementary Material S3.

### 2.2. Design

This study employed an exploratory research design to gain a comprehensive understanding of participants’ perceptions and experiences regarding the perceived effectiveness of HIV prevention programmes in the O.R. Tambo District Municipality. As the aim was to gather detailed insights rather than test hypotheses, this design emphasises exploration and the development of a deeper understanding of the issue [19,21].

The exploratory approach was appropriate for this study because it focused on participants’ experiences, perceptions, and views of the perceived effectiveness of these programmes. It enabled the collection of rich, detailed data, which is essential for uncovering the underlying factors that influence engagement. By adopting this approach, the study identified key issues affecting the utilisation of HIV prevention services and provided a more thorough understanding of the topic [21].

### 2.3. Setting

The study was conducted in two public primary healthcare clinics providing HIV prevention services within the O.R. Tambo District Municipality in the Eastern Cape Province of South Africa. To protect the identities of the facilities, the clinics are referred to as Clinic A and Clinic B. Clinic A is situated in a predominantly rural area, while Clinic B is located in an urban setting within the district.

The clinics were purposively selected because they provide a comprehensive range of HIV prevention services, including HIV testing services, condom distribution, pre-exposure prophylaxis (PrEP), and prevention of mother-to-child transmission (PMTCT). In addition, the facilities serve communities with a high HIV burden and represent diverse service-delivery contexts within the district. The inclusion of both rural and urban clinics enabled the study to capture a broader range of experiences and perspectives regarding the perceived effectiveness of HIV prevention programmes.

### 2.4. Population

The study involved two main participant groups. The first group consisted of clients aged 18 years and older who accessed services at the selected clinics. The second group comprised healthcare professionals directly involved in delivering HIV prevention programmes. These included professional nurses, lay counsellors, peer educators, health promoters, and pharmacy personnel who participated in focus group discussions.

These participants were selected for their direct involvement in both the provision and utilisation of HIV prevention services. Including both groups allowed the study to capture a balanced understanding of the programmes by considering both user experiences and professional perspectives.

### 2.5. Sampling Method and Techniques

This study employed a nonprobability sampling method, specifically purposive sampling. Purposive sampling involves the intentional selection of participants based on specific characteristics that are relevant to the research topic [22,23]. This approach is particularly useful when the aim is to obtain in-depth insights from individuals with knowledge or experience related to the phenomenon under study [23].

Participants were selected based on their direct involvement in HIV prevention programmes, either as clients who accessed these services or as healthcare professionals responsible for delivering them. This method enabled the researchers to focus on individuals well-positioned to provide detailed and meaningful information. As a result, the study generated rich, relevant data on the perceived effectiveness of HIV prevention programmes.

### 2.6. Sample Size

The study included 15 clients who participated in semi-structured interviews and 10 healthcare professionals who took part in focus group discussions. The healthcare professionals comprised nurses (professional, enrolled nurse assistant, and enrolled nurse) (*n* = 4), lay counsellors (*n* = 2), peer educators (*n* = 2), health promoters (*n* = 1), and pharmacy personnel (*n* = 1). Including participants from these different professional categories enabled the study to capture a broad range of perspectives on the implementation and perceived effectiveness of HIV prevention programmes.

The use of both individual interviews and focus group discussions enabled comparisons of perspectives across different participant groups and settings. All participants were aged 18 or older. Including both clients and healthcare professionals ensured that the study captured perspectives from both service users and service providers. This combination contributed to a more balanced and comprehensive interpretation of the findings.

Information saturation was achieved by the 15th client interview. Saturation was assessed concurrently with data collection and analysis through ongoing review of interview transcripts and emerging codes. As data collection progressed, the researchers monitored the recurrence of ideas, concepts, and experiences across participants. Saturation was considered achieved when successive interviews and focus group discussions yielded no substantially new codes, categories, or insights relevant to the research objectives, and the existing themes were sufficiently developed to answer the research question. The assessment of saturation was guided by recommendations for qualitative research, which define saturation as the point at which additional data no longer yield new information relevant to the study’s aims [22,23].

Participants were selected based on their regular use of HIV prevention services and their willingness to share their experiences. Individuals under 18 years of age were excluded from the study. The demographic characteristics of the participants are presented in Tables S1 and S2. Table S1 summarises the characteristics of client participants, while Table S2 presents the characteristics of healthcare professionals who participated in the focus group discussions.

To maintain confidentiality while providing contextual information, participants were assigned identification codes. For clients, codes comprised the participant number (P), gender (F = Female, M = Male, NB = Non-binary), and age in years. For example, P3F25 refers to Participant 3, Female, aged 25 years. Healthcare professionals were assigned codes based on their professional roles: Professional Nurse (HCP-PN), Enrolled Nurse Assistant (HCP-ENA), Enrolled Nurse (HCP-EN, Lay Counsellor (HCP-LC), Peer Educator (HCP-PE), Health Promoter (HCP-HP), and Pharmacy Assistant (HCP-PA). These codes were used consistently throughout the presentation of findings to protect participant anonymity while indicating the source of quotations.

### 2.7. Data Collection

Data were collected through semi-structured interviews with clients and focus group discussions with healthcare professionals. Semi-structured interviews are a qualitative data collection method that uses open-ended questions to explore participants’ perspectives in depth [24]. This approach allows the researchers to ask follow-up questions where necessary, enabling a more detailed understanding of participants’ views and experiences [25]. The flexibility of this method made it particularly suitable for the study, enabling a comprehensive exploration of participants’ experiences with HIV prevention programmes.

In addition, focus group discussions were conducted with healthcare professionals. A focus group is a qualitative method that generates data through guided group interaction, allowing participants to share and reflect on their experiences collectively [26,27]. This approach is especially useful for examining attitudes, beliefs, and practices, as it captures both individual responses and shared perspectives that emerge through discussion [26]. One focus group discussion was conducted with ten healthcare professionals drawn from the two participating clinics.

Both semi-structured interviews and focus group discussions involved engaging participants in guided conversations about the perceived effectiveness of HIV prevention programmes in the O.R. Tambo District Municipality. The combination of these methods strengthened the study by providing rich, detailed data and enabling a deeper understanding of both individual experiences and group perspectives.

All interviews and focus group discussions were audio-recorded with participants’ permission to ensure accurate capture of information. No video recordings were made. Interviews were conducted in locations that ensured privacy and confidentiality. Individual interviews lasted 30 to 45 min, while focus group discussions lasted 60 to 90 min. Field notes were taken during and immediately after each interview and discussion to document contextual observations and non-verbal communication that could assist interpretation in the analysis.

Data collection was conducted primarily in isiXhosa and English, using semi-structured interviews with clients. Focus group discussions with healthcare professionals were conducted in English. Audio recordings were transcribed verbatim by the principal researcher. Interviews conducted in isiXhosa were translated into English by the principal researcher, who is proficient in both isiXhosa and English. To ensure accuracy and preserve the intended meaning of participants’ responses, the translated transcripts were reviewed against the original audio recordings by the researcher and subsequently checked by the supervisor during the analysis process.

#### Research Team and Reflexivity

Data collection was conducted by the principal researcher, a female Master’s student with training in qualitative research methods under the supervision of a PhD holder. The researcher had no prior relationship with the participants before recruitment. Participants were informed of the researcher’s academic role, the aims of the study, and the purpose of the research before participation. Reflexive notes were maintained throughout the study to minimise the influence of researcher assumptions during data collection and analysis.

### 2.8. Trustworthiness

Trustworthiness in qualitative research refers to the extent to which the findings are credible, transferable, dependable, and confirmable, thereby ensuring the rigour and overall quality of the study [28,29].

Credibility was enhanced by selecting participants with direct experience with HIV prevention programmes and by using multiple data collection methods, namely semi-structured interviews and focus group discussions, to obtain rich, detailed information. Transferability was supported by a clear, detailed description of the study context, setting, and participant characteristics, enabling readers to assess the extent to which the findings may apply to similar contexts.

Dependability was ensured through a consistent, systematic research process, including semi-structured interview guides and clearly defined data-collection procedures. Finally, confirmability was achieved by maintaining objectivity throughout the research process, ensuring that the findings were grounded in participants’ responses rather than personal bias, and by keeping thorough records and reflections during data analysis.

Member checking was conducted by providing selected participants with opportunities to verify the accuracy of transcripts and clarify statements where necessary. This process enhanced the credibility of the findings.

### 2.9. Data Analysis

This study employed thematic analysis to examine the qualitative data, guided by the framework proposed by Braun and Clarke [30]. Thematic analysis is a flexible approach used to identify, analyse, and report patterns or themes within qualitative data [30].

The researchers followed the six phases of thematic analysis outlined by Braun and Clarke [30]. The process began with familiarisation, during which the researcher repeatedly read the transcribed interviews and focus group discussions to develop a thorough understanding of the data. This was followed by generating initial codes across the dataset, during which meaningful segments of data relevant to the research objectives were identified.

The codes were then grouped into potential themes. These themes were reviewed and refined to ensure that they accurately represented the data. During this stage, each theme was carefully defined, with attention given to its relevance and contribution to the research questions. Themes were then clearly named and described.

To strengthen the credibility of the findings, the themes were supported by direct quotations from participants. Finally, the themes were organised in relation to the study’s objectives and research questions, and a comprehensive report was developed to present the findings.

Data coding was conducted by the principal researcher, while thematic development and interpretation were reviewed and discussed with the supervisor to enhance analytical rigour.

The findings and discussion are presented in an integrated thematic format. This approach was adopted to facilitate the simultaneous presentation and interpretation of participants’ views alongside relevant literature and the Health Belief Model. Integrating findings and discussion enabled the researchers to contextualise emerging themes within existing empirical evidence and theoretical perspectives, thereby providing a richer and more comprehensive understanding of factors influencing the perceived effectiveness of HIV prevention programmes.

Data management and analysis were conducted manually without the use of qualitative analysis software.

### 2.10. Ethical Considerations

Ethical clearance for this study was obtained from a higher education institution in the Eastern Cape (Ethics Number: WSUHREC186/2025), approved on the 11 August 2025. Permission to conduct the research was also granted by the Eastern Cape Department of Health (ECDoH) and the selected clinics within the O.R. Tambo District Municipality. The study was conducted in line with established ethical principles for research involving human participants.

Informed consent was obtained from all participants after they had been provided with clear and detailed information about the purpose of the study, the procedures involved, and any potential risks and benefits. Participation was entirely voluntary, and participants were informed of their right to withdraw from the study at any stage without any negative consequences.

Confidentiality and privacy were strictly maintained throughout the research process. All data were anonymised, and identifying information was removed to ensure that responses could not be traced back to individual participants. Access to the data was limited to the principal researcher, and all information was securely stored to protect participants’ privacy and uphold ethical standards.

## 3. Findings and Discussion

The following themes were generated from the empirical data collected through semi-structured interviews with clients and focus group discussions with health care professionals:

### 3.1. Theme 1: Perceived Effectiveness of HIV Prevention Programmes Depends on Adherence and Correct Use

The findings of this study indicate that both clients and healthcare professionals perceive HIV prevention programmes—particularly condom use and pre-exposure prophylaxis (PrEP)—as effective, but only when they are used consistently and correctly. This highlights that effectiveness is not simply an inherent feature of the intervention itself, but rather depends on how it is used in practice.

Participants consistently pointed out that non-adherence, discontinuation, and incorrect use reduce the effectiveness of these programmes. This shifts the understanding of effectiveness from being purely biomedical to one that is also shaped by behavioural and contextual factors. In this way, the success of HIV prevention programmes is closely linked to individuals’ actions, perceptions, and circumstances. The following excerpts from clients illustrate these perspectives:

*“… I would say they [prevention programmes] would have been 100% effective if I was adhering… because sometimes we do not follow the instructions properly or we default, and that is when we start doubting whether they work or not.”*

(P3F25)

*“They are effective when you follow all the instructions given. I have seen other people saying that it is not effective and you can still get the virus, but I believe you only get the virus because you did not follow the procedure correctly… either a person defaulted or they were not consistent.”*

(P11NB23)

*“The HIV prevention programmes are effective if you follow them. If you use condoms, if you take PrEP or PEP as guided, they are effective… and with the PrEP injection it is even more effective and convenient for me.”*

(P8M25)

On the other hand, the focus group discussion also confirms the clients’ notion. The following were the responses:

*“The prevention programmes can be very effective… when people are adhering to ART, they suppress and reduce the chances of infecting others. If they are using condoms consistently, they are protected, and PrEP when taken consistently is very effective.”*

(HCP-LC1)

*“… I believe it is effective and it can be more effective if the clients could adhere… we initiate them on pills and they come for the second month but sometimes they never come back and end up defaulting.”*

(HCP-LC2)

*“… If people were adherent to the treatment, I feel that they would be 99% effective when it comes to minimising the infection rate… but some are avoiding these programmes.”*

(HCP-PN1)

These findings are strongly supported by existing empirical evidence, which shows that adherence is a key factor in determining the effectiveness of HIV prevention interventions [31]. For instance, a systematic review by [32] found that high discontinuation rates and suboptimal adherence significantly reduce the real-world effectiveness of oral PrEP, with nearly half of users discontinuing within the first 6 months. Similarly, ref. [33] reported that the protective benefits of PrEP are closely linked to consistent use, with markedly higher effectiveness among individuals who adhere regularly compared to those who use it inconsistently. These findings are consistent with participants’ accounts in this study, where inconsistent use was associated with doubts about the effectiveness of prevention methods.

Further support comes from longitudinal cohort studies, which highlight the importance of sustained adherence over time. Ref. [34] identified different adherence patterns among PrEP users and showed that individuals with consistently high adherence had significantly lower rates of HIV infection compared to those with declining or minimal use. This aligns with the perspectives of healthcare professionals in the current study, who emphasised that programme effectiveness improves when clients maintain regular and consistent use of prevention strategies.

The findings also reflect the widely recognised “efficacy–effectiveness gap” in HIV prevention. While clinical trials often demonstrate high efficacy under controlled conditions, outcomes in real-world settings are frequently influenced by behavioural and structural challenges. For example, ref. [35] found that long-acting injectable cabotegravir (CAB-LA) was more effective than daily oral PrEP, largely because it reduces the need for daily adherence. The introduction of long-acting prevention methods, therefore, offers a promising approach to addressing adherence-related challenges. This perspective was also evident in participants’ responses, where injectable PrEP was described as more convenient and easier to maintain.

However, even with these advances in biomedical prevention, adherence remains a persistent concern. Evidence from a randomised trial [36] conducted among young women in South Africa showed that adherence to PrEP remains low in the absence of targeted support interventions. This suggests that behavioural and social factors continue to play a significant role in shaping effective use. Similarly, real-world cohort data from [37] indicate that adherence is influenced by a range of individual and contextual factors, including side effects, perceived risk, and social influences. These findings highlight the complexity of maintaining consistent use over time.

Taken together, the evidence suggests that the perceived effectiveness of HIV prevention programmes cannot be assessed solely on the basis of availability or biomedical efficacy. Rather, perceived effectiveness should be understood as a dynamic outcome shaped by adherence behaviours, individual decision-making, and broader contextual influences. The alignment between participants’ views and the existing literature reinforces the importance of integrating behavioural support, adherence counselling, and context-specific strategies into HIV prevention efforts.

Therefore, while HIV prevention programmes are widely recognised as effective, their real-world impact depends largely on sustained, correct use. Addressing adherence challenges is essential to maximise programme effectiveness and achieve meaningful reductions in HIV transmission.

From the HBM perspective, these findings reflect the constructs of perceived benefits and self-efficacy. Participants generally believed that HIV prevention methods such as PrEP and condoms are effective when used correctly, indicating strong perceptions of benefit. At the same time, their accounts demonstrate that confidence in one’s ability to adhere consistently to prevention strategies is critical to achieving the desired outcomes. Thus, effectiveness is influenced not only by the availability of interventions but also by individuals’ self-efficacy to use them appropriately and consistently.

### 3.2. Theme 2: Structural, Social and Individual Barriers Limit Programme Effectiveness

Although participants recognised the perceived effectiveness of HIV prevention programmes, they also highlighted several factors that limit their overall impact. These included stigma, concerns about side effects, misinformation, and various structural challenges. Such factors were perceived as significant barriers that affect the consistent use and acceptance of these interventions.

Participants explained that negative social attitudes and fear of being judged often discourage individuals from accessing or openly using prevention services. In addition, concerns about possible side effects, particularly with PrEP, were reported to influence decisions to discontinue or avoid use. Misinformation and a lack of accurate knowledge further contributed to uncertainty and reduced confidence in these programmes. Structural challenges, such as limited access to services and resource constraints, also hindered effective utilisation. The following excerpts from clients illustrate these concerns:

*“… I feel like when I go to the clinic most of the time the staff is not professional… I get embarrassed and feel unsupported and judged when I am taking my medication. That makes it difficult to continue.”*

(P3F25)

*“… One of the reasons why I did not take it correctly was because of the changes it made to my body… like vaginal itchiness, and it was embarrassing especially around people.”*

(P4F24)

*“You know with the stigma around HIV, not everyone understood why I opted for PrEP… it was perceived as if I am now going to be careless, and that made me feel misunderstood.”*

(P10F21)

*“… Sometimes when I go there, I find there was no stock… and I would be told to go to another facility, and because I do not want to use the clinic near my house, it becomes difficult.”*

(P3F25)

The participants from the focus group discussion responded:

*“Clients will gladly start the treatment but they are never consistent… they complain about side effects, and some end up stopping the treatment completely and that is a big problem we are facing.”*

(HCP-PN2)

*“The problem is that some clients refuse PrEP… they say they already know they will get side effects like nausea or diarrhoea, even before trying it.”*

(HCP-LC1)

*“Many females do not use prevention methods because of their partners… they are afraid to say no to their partners, so that also affects how effective these programmes are.”*

(HCP-ENA)

*“From the pharmaceutical side, the unavailability of stock is a challenge…”*

(HCP-PA1)

The findings of this study show that the perceived effectiveness of HIV prevention programmes is significantly limited by a combination of structural, social, and individual barriers. Participants identified stigma, side effects, misinformation, and health system challenges—such as stock shortages and limited accessibility—as key factors that affect the consistent use of prevention methods. These findings suggest that, although effective biomedical interventions are available, their impact in real-world settings is often shaped by contextual and behavioural influences.

Stigma emerged as a major barrier affecting both the uptake and continued use of prevention services. Participants reported experiencing judgment in healthcare settings and communities, which discouraged them from seeking or maintaining engagement with these services. This is consistent with [38], who found that both anticipated and experienced stigma—particularly the association of PrEP use with HIV-positive status or perceived promiscuity—can negatively influence decision-making and adherence among young women. Similarly, ref. [39] reported that HIV-related stigma remains widespread in many African contexts and continues to limit access to both prevention and treatment services. These findings indicate that stigma operates at multiple levels, shaping how individuals perceive and respond to HIV prevention interventions.

In addition to stigma, individual-level factors such as side effects and perceived changes in health were reported to disrupt adherence. Participants described experiences of nausea, headaches, and general discomfort, which contributed to inconsistent use or discontinuation of prevention methods. This is supported by [40], who found that side effects and bodily changes are significant reasons for interruptions in PrEP use among young women in South Africa. In many cases, even the anticipation of side effects was enough to discourage individuals from starting or continuing with prevention methods.

Structural and health system challenges further complicated access to and use of these services. Participants highlighted issues such as medication stock-outs, long waiting times, and the need to travel long distances to reach healthcare facilities. These barriers disrupt continuity of care and make it more difficult for individuals to maintain consistent use of prevention strategies. Similar findings have been reported by [41], who emphasise that weak health systems, fragmented service delivery, and limited resources in many African settings undermine the effectiveness of HIV prevention programmes. Ref. [42] also identifies factors such as healthcare provider attitudes, limited service availability, and lack of social support as key barriers affecting both the initiation and sustained use of PrEP.

Taken together, these findings show that the perceived effectiveness of HIV prevention programmes cannot be understood solely in terms of biomedical efficacy. Instead, it is shaped by a range of interconnected social, behavioural, and system-level factors. The consistency between participants’ experiences and existing evidence underscores the need for more comprehensive approaches that go beyond service provision. Efforts to reduce stigma, improve user experiences, and strengthen health systems are essential. Without addressing these challenges, the full potential of HIV prevention programmes to reduce transmission is unlikely to be realised.

The barriers identified in this study align closely with the HBM construct of perceived barriers. Participants described stigma, fear of side effects, misinformation, unsupportive healthcare experiences, and medication stock shortages as obstacles that limit engagement with HIV prevention services. According to the HBM, even when individuals recognise the benefits of preventive actions, significant barriers may reduce the likelihood of adopting or maintaining those behaviours. The findings suggest that reducing these barriers is essential for improving programme utilisation and effectiveness.

### 3.3. Theme 3: Accessibility and Availability Influence Perceived Effectiveness

Participants generally viewed HIV prevention services as available; however, they emphasised that inconsistencies in access, availability, and service delivery undermine their overall effectiveness. While services may exist, challenges in consistent provision and reliable access limit their sustained use and impact. The following excerpts from clients reflect these experiences:

*“… In my community it is easy to get condoms because they are everywhere… you go to shops, taverns, even at the clinic you just take them without waiting in line.”*

(P2F28)

*“I could say it is easy because when I go for family planning, I also ask for PrEP… they give me the services at the same time.”*

(P12F19)

*“It is not too difficult but not too easy… sometimes there is no stock, and you are told to go to another facility, and that makes it complicated.”*

(P3F25)

*“It is not easy finding a facility where they have the injection… most facilities still have pills, and that limits options.”*

(P12F19)

The health care professionals also asserted:

*“Clients do not like coming to the clinic… they complain about financial constraints, transport and time. It becomes more effective when we bring services to them through mobile clinics.”*

(HCP-LC1)

*“Our facility only has oral PrEP… we do not yet have the injection, and that limits what we can offer.”*

(HCP-PN)

*“When services are provided closer to communities, like outreach or mobile services, people are more consistent and the programmes become more effective.”*

(HCP-LC2)

The findings of this study show that the accessibility and availability of HIV prevention services play an important role in shaping their use and overall perceived effectiveness. Participants identified several challenges, including long distances to healthcare facilities, limited availability of services, and inconsistent access to prevention commodities. These factors were seen to restrict engagement with prevention programmes. This suggests that even when interventions are present, their effectiveness is reduced if they are not easily accessible to the populations they are intended to serve.

Existing research supports the importance of geographical and structural barriers in influencing the use of HIV prevention services. Ref. [43] found that many individuals in sub-Saharan Africa live in areas with limited access to nearby healthcare facilities, and that distance and travel time significantly affect service utilisation. Similarly, ref. [44] reported that the uptake and continued use of PrEP among adolescent girls and young women remain low despite its availability, largely due to challenges related to access and service delivery.

In addition to physical access, differences in service provision further limit utilisation. Ref. [45] highlights a significant gap between the need for PrEP and its actual delivery across various settings, indicating that many high-risk groups remain underserved. These inequalities are further influenced by broader systemic challenges, such as limited infrastructure, insufficient resources, and delays in implementing new prevention strategies.

At the same time, emerging evidence suggests that improving access through more flexible and user-centred approaches can increase engagement with HIV prevention services. Ref. [46] found that decentralised and community-based service models improve access, user satisfaction, and uptake by responding more effectively to individual and contextual needs.

Overall, these findings demonstrate that accessibility and availability are central to the perceived effectiveness of HIV prevention programmes. Addressing geographical, structural, and service delivery challenges is therefore essential to improve equitable access and maximise the impact of these interventions.

These findings can also be interpreted through the HBM framework. Difficulties with service accessibility and availability constitute practical barriers that hinder preventive behaviour. Conversely, outreach programmes, mobile clinics, and integrated service delivery function as cues to action by making prevention services more visible, convenient, and accessible. Improving access, therefore, not only reduces barriers but may also increase individuals’ motivation to engage in HIV prevention behaviours.

### 3.4. Theme 4: Need for Improved Programme Delivery to Enhance Effectiveness

Both participant groups emphasised the need to improve education, reduce stigma, strengthen health systems, and adopt innovative approaches in order to enhance the effectiveness of HIV prevention programmes. These factors were viewed as essential for addressing the challenges that limit consistent use and engagement with prevention services.

Participants highlighted that increased awareness and accurate information could help to correct misconceptions and promote informed decision-making. At the same time, efforts to reduce stigma were seen as critical in encouraging individuals to access and continue using these services without fear of judgment. Strengthening health systems, including improving service delivery and ensuring consistent availability of resources, was also identified as an important step towards improving programme outcomes. In addition, participants highlighted the value of introducing innovative approaches, such as new prevention methods and more accessible service models, to better meet the needs of diverse populations. The following excerpts from clients illustrate these perspectives:

*“… There is not enough education around these programmes… most people are not informed, and some do not even know the difference between PrEP and PEP.”*

(P7M34)

*“I think social media would play a huge role because people use it as a source of information… if it is used properly it can reach a lot of people.”*

(P9F22)

*“Reducing stigma is very important because when we feel accepted, we are more likely to access and use these services.”*

(P8M25)

*“Creating safe spaces… support groups and environments where people are not judged would make it easier for people to engage.”*

(P5F33)

The health care professionals also asserted:

*“We need stronger government leadership and adequate resources… nurses are overworked, and it affects how services are delivered.”*

(HCP-PA1)

*“If the department could employ more healthcare professionals, it would reduce workload and allow us to support patients better.”*

(HCP-PE1)

*“We rely on NGOs for many programmes… when funding ends, those services stop, and that affects continuity and effectiveness.”*

(HCP-EN)

The findings of this study highlight that knowledge and awareness play a central role in influencing the uptake and effective use of HIV prevention programmes. Participants displayed varying levels of understanding of different prevention methods, with misconceptions and limited awareness contributing to inconsistent use and reduced engagement. This indicates that mere service availability is not sufficient; individuals also need adequate knowledge to make informed decisions about prevention.

Existing literature supports the view that knowledge is a key factor in the uptake of HIV prevention strategies. Ref. [47] found that awareness of PrEP among women in sub-Saharan Africa remains low, with fewer than 15% demonstrating sufficient knowledge, which significantly limits its utilisation. Similarly, ref. [48] reported a strong relationship between awareness and uptake, noting that individuals who are better informed about prevention options are more likely to access and use them.

Community-based education and mobilisation efforts have also been shown to improve engagement with HIV prevention services. Ref. [49] demonstrated that such interventions can increase HIV testing and linkage to care by addressing social barriers and strengthening awareness. In addition, peer-led education initiatives have proven effective in improving both knowledge and behaviour. For example, ref. [50] reported notable improvements in HIV-related knowledge following the implementation of peer education programmes.

Furthermore, community-led approaches that integrate education with service delivery show promising outcomes in increasing participation. Ref. [51] found that community-driven HIV self-testing initiatives enhanced awareness and uptake, particularly in settings where access to facility-based services is limited.

Overall, these findings demonstrate that knowledge and awareness are essential to the success of HIV prevention programmes. Strengthening health education, addressing misconceptions, and adopting community- and peer-based approaches are important steps towards improving uptake and ensuring individuals can use available prevention methods effectively.

Theme 4 reflects several HBM constructs, particularly cues to action, perceived benefits, and self-efficacy. Educational campaigns, peer support, social media initiatives, and community outreach activities serve as cues that encourage uptake of HIV prevention services. Improved knowledge increases awareness of the benefits associated with prevention strategies, while supportive health systems and stigma-free environments enhance individuals’ confidence in their ability to adopt and sustain preventive behaviours. These findings underscore the importance of interventions that strengthen both motivation and capability to engage with HIV prevention programmes.

## 4. Recommendations

To enhance the effectiveness of HIV prevention programmes, a multi-level and integrated approach is required. This approach should address individual, social, and systemic factors that influence the uptake and consistent use of prevention strategies.

Strengthening adherence support is essential. This can be achieved by providing ongoing counselling and follow-up for individuals using PrEP and condoms. In addition, introducing more user-friendly options, such as long-acting PrEP, may improve consistency in use. The use of digital tools, such as SMS reminders and mobile applications, can further support adherence by helping individuals maintain consistent prevention practices.

Addressing stigma and social barriers is equally important. Community-based stigma reduction campaigns can help challenge negative attitudes and misconceptions surrounding HIV. At the same time, healthcare professionals should be trained to provide non-judgmental and youth-friendly services. Efforts should also be made to ensure that services are inclusive and responsive to the needs of vulnerable and key populations.

Improving accessibility and service delivery is critical for increasing utilisation. Expanding community-based and mobile HIV services can bring prevention closer to those in need, particularly in underserved areas. Decentralising services is especially important for reaching rural populations. Furthermore, ensuring a consistent supply of prevention commodities, such as condoms and PrEP, is necessary to support uninterrupted access.

Enhancing health education and awareness can significantly improve engagement with prevention programmes. Targeted education initiatives should focus on addressing misconceptions and providing accurate, relevant information. Peer education and community outreach programmes can also play a key role in increasing awareness and encouraging behavioural change. In addition, integrating HIV education into schools, universities, and broader public campaigns can promote sustained awareness.

Finally, strengthening health systems is fundamental to supporting all other interventions. This includes improving resource allocation and strengthening supply chain management to avoid service disruptions. Integrating HIV prevention with other health services, such as sexual and reproductive healthcare, can also improve efficiency and accessibility. Investing in sustainable, person-centred service delivery models is essential to ensure long-term effectiveness.

## 5. Limitations

This study should be interpreted in light of several limitations. Firstly, the study was conducted in two selected clinics within the O.R. Tambo District Municipality, and the findings may therefore not be transferable to other districts, provinces, or healthcare settings with different social, cultural, and health system contexts. The purpose of the study was to obtain an in-depth understanding of participants’ experiences rather than to produce statistically generalisable findings.

Secondly, the study relied on self-reported information from clients and healthcare professionals. As a result, some participants may have provided socially desirable responses, particularly when discussing sensitive issues such as HIV-related stigma, adherence to prevention programmes, and sexual health behaviours. This may have influenced the nature of the information shared during interviews and focus group discussions.

Thirdly, data from healthcare professionals were collected through focus group discussions. Although this approach encouraged interaction and the sharing of experiences, some participants may have been reluctant to express views that differed from their colleagues’, which may have limited the range of perspectives captured.

The study also employed purposive sampling to recruit participants with direct experience of HIV prevention programmes. While this approach was appropriate for achieving the study objectives, it may have excluded individuals with different experiences or views, thereby limiting the diversity of perspectives represented in the findings.

In addition, the researchers acknowledge that personal experiences, assumptions, and interpretations may have influenced aspects of data collection and analysis. To minimise this possibility, the study followed a systematic research process and maintained reflexive awareness throughout.

Finally, client interviews were conducted in participants’ preferred language (isiXhosa or English), while focus group discussions with healthcare professionals were conducted in English. Interviews conducted in isiXhosa were translated into English for analysis and reporting. Although careful procedures were followed to preserve meaning during transcription and translation, the possibility of subtle differences in interpretation cannot be entirely excluded.

Despite these limitations, several measures were implemented to enhance the study’s trustworthiness, including the use of multiple participant groups, systematic thematic analysis, and detailed documentation of the research process.

## 6. Conclusions

This study set out to explore the perceived effectiveness of HIV prevention programmes. The findings show that perceived effectiveness is shaped by a complex interaction of behavioural, structural, and informational factors. Across the four key themes, it is evident that although strategies such as condom use and PrEP are widely recognised as effective, their impact in practice remains conditional.

Firstly, effectiveness depends heavily on consistent, correct use, underscoring the importance of adherence. Secondly, a range of barriers—including stigma, concerns about side effects, and limitations within the health system—continue to affect the success of these programmes. Thirdly, challenges related to accessibility and availability limit utilisation, particularly among individuals in rural or underserved communities. Lastly, gaps in knowledge and awareness, as well as persistent misconceptions, reduce individuals’ ability to make informed decisions and engage effectively with prevention methods.

Overall, these findings suggest that HIV prevention programmes cannot be evaluated based on biomedical efficacy alone. Instead, their perceived effectiveness should be understood in relation to how individuals interact with these interventions within their social, cultural, and structural contexts. Without addressing challenges related to adherence, unequal access, and limited awareness, the full potential of these programmes is unlikely to be realised.

While this study provides important insights into participants’ experiences and perspectives, the findings should be interpreted within the context of its geographic and methodological scope. Specifically, the study was conducted in two clinics within a single district and employed a qualitative research design; therefore, the results are not intended to be statistically generalisable but may be transferable to similar primary healthcare contexts. Future studies across multiple districts and settings, using complementary methodological approaches, could further strengthen and extend these findings.

## Data Availability

The raw data supporting the conclusions of this article will be made available by the authors on request.

## Supplementary Materials

The following supporting information can be downloaded at: https://www.mdpi.com/article/10.3390/idr18050114/s1, Table S1: Demographic Characteristics of Client Participants; Table S2: Demographic Characteristics of Healthcare Professional Participants; Supplementary Material S3: Consolidated Criteria for Reporting Qualitative Research (COREQ) Checklist.

## Abbreviations

The following abbreviations are used in this manuscript:

AIDS	Acquired Immunodeficiency Syndrome
CD4	Cluster of Differentiation 4 (T-helper cells)
ECDoH	Eastern Cape Department of Health
HIV	Human Immunodeficiency Virus
PrEP	Pre-Exposure Prophylaxis
PMTCT	Prevention of mother-to-child transmission
WHO AFRO	World Health Organization The World Health Organization African Region
