# Supplementary material for: The Views of Clients and Healthcare Professionals on the Perceived Effectiveness of HIV Prevention Programmes in Selected O. R. Tambo District Municipality Clinics
Source: Infect Dis Rep. 2026 Oct 7;18(5):114. doi: 10.3390/idr18050114 (PMC13704696; doi:10.3390/idr18050114)
Supplement: Supplementary file 1 [file idr-18-00114-s001.zip › idr-4401234 - supplementary-Table S1 & S2 Demographics of participants.pdf]

Table S1.Demographic Characteristics of Client Participants

Pseudonyms	Gender	Age
P1F27	Female	27
P2F28	Female	28
P3F25	Female	25
P4F24	Female	24
P5F33	Female	33
P6F21	Female	21
P7M34	Male	34
P8M25	Male	25
P9F22	Female	22
P10F21	Female	21
P11NB23	Non-Binary	23
P12NB19	Female	19
P13M20	Non-binary	20
P14M29	Male	29
P15M18	Male	18

Table S2. Demographic Characteristics of Healthcare Professional Participants

Pseudonyms	Age and gender	Profession	Experience
HCP-PN1	30 - Female	Professional Nurse	7years
HCP-PA1	40- Female	Pharmacist Assistant	13 years
HCP-ENA	35- Female	Enrolled nurse assistant	2 years
HCP-PE1	29- Female	Peer educator	4 years
HCP-LC1	27- Female	Lay counsellor	5years
HCP-HP	32- Female	Health promoter	3years
HCP-PN2	46- Female	Professional nurse	15 years
HCP-PE	26- Female	Peer educator	7 years
HCP-LC2	30- Female	Lay counsellor	10 years
HCP- EN	41- Female	Enrolled nurse	11 years
