# Supplementary material for: The Views of Clients and Healthcare Professionals on the Perceived Effectiveness of HIV Prevention Programmes in Selected O. R. Tambo District Municipality Clinics
Source: Infect Dis Rep. 2026 Oct 7;18(5):114. doi: 10.3390/idr18050114 (PMC13704696; doi:10.3390/idr18050114)
Supplement: Supplementary file 1 [file idr-18-00114-s001.zip › idr-4401234- COREQ Checklist.pdf]

Supplementary Material S3. COREQ Checklist

Manuscript title: *The Views of Clients and Healthcare Professionals on the Perceived Effectiveness of HIV Prevention Programmes in Selected O.R. Tambo District Municipality Clinics*

Domain 1: Research team and reflexivity

Personal characteristics

1. Interviewer

Reported? Yes

Description: The principal researcher conducted the semi-structured interviews and focus group discussions.

Location: Section 2.7.1, page 6-7

2. Credentials

Reported? Yes

Description: The researcher was a Master's student trained in qualitative research methods under supervision.

Location: Section 2.7.1

3. Occupation

Reported? Yes

Description: Master's student/researcher.

Location: Section 2.7.1

4. Gender

Reported? Yes

Description: Female researcher.

Location: Section 2.7.1

5. Experience and training

Reported? Yes

Description: Researcher received training in qualitative research methods and conducted the study under supervision.

Location: Section 2.7.1

Relationship with participants

6. Relationship established

Reported? Yes

Description: No prior relationship existed between the researcher and participants before recruitment.

Location: Section 2.7.1

7. Participant's knowledge of the interviewer

Reported? Yes

Description: Participants were informed about the researcher's academic role, study purpose, and objectives.

Location: Section 2.7.1

8. Interviewer characteristics

Reported? Yes

Description: Reflexive notes were maintained throughout data collection and analysis to minimise researcher influence.

Location: Section 2.7.1

Domain 2: Study design

Theoretical framework

9. Methodological orientation

Reported? Yes

Description: Exploratory qualitative study guided by the Health Belief Model and analysed using thematic analysis following Braun and Clarke.

Location: Sections 1.3, 2.1, 2.2, and 2.9

Participant selection

10. Sampling

Reported? Yes

Description: Purposive sampling.

Location: Section 2.5

11. Method of approach

Reported? Partially

Description: Participants were purposively recruited based on direct involvement in HIV prevention services.

Location: Section 2.5

12. Sample size

Reported? Yes

Description: 15 clients and 10 healthcare professionals.

Location: Section 2.6

13. Non-participation

Reported? No

Setting

14. Setting of data collection

Reported? Yes

Description: Two public primary healthcare clinics (one rural and one urban) in the O.R. Tambo District Municipality.

Location: Section 2.3

15. Presence of non-participants

Reported? No

16. Description of sample

Reported? Yes

Description: Demographic characteristics provided in supplementary Tables S1 and S2.

Location: Section 2.6

Data collection

17. Interview guide

Reported? Yes

Description: Semi-structured interview and focus-group discussion guides were used.

Location: Section 2.7

18. Repeat interviews

Reported? No

19. Audio/visual recording

Reported? Yes

Description: Audio recordings were made with participant consent.

Location: Section 2.7

20. Field Notes

Reported? Yes

Description: Field notes were taken during and after interviews and focus groups.

Location: Section 2.7

21. Duration

Reported? Yes

Description: Interviews: 30-45 minutes. FGDs: 60-90 minutes.

Location: Section 2.7

22. Data saturation

Reported? Yes

Description: Information saturation achieved by the 15th client interview.

Location: Section 2.6

23. Transcripts returned

Reported? Yes

Description: Selected participants were given the opportunity to verify transcripts and clarify information.

Location: Section 2.8

Domain 3: Analysis and findings

Data analysis

24. Number of data coders

Reported? Yes

Description: The principal researcher conducted coding, while thematic development and interpretation were reviewed with the supervisor.

Location: Section 2.9

25. Description of coding tree

Reported? No

26. Derivation of themes

Reported? Yes

Description: Themes were derived inductively through thematic analysis.

Location: Section 2.9

27. Software

Reported? No

Description: Data management and analysis were conducted manually without the use of qualitative analysis software.

Reported? Yes

Location: Section 2.9

28. Participant checking

Reported? No

Description: Participants did not provide feedback on the findings, themes, or interpretation of the data.

Location: Section 2.8

Reporting

29. Quotations presented

Reported? Yes

Description: Participant quotations presented for all themes.

Location: Section 3

30. Data and findings consistent

Reported? Yes

Description: Findings supported by participant quotations.

Location: Section 3

31. Clarity of major themes

Reported? Yes

Description: Four major themes were clearly identified and discussed.

Location: Section 3

32. Clarity of minor themes

Reported? Yes

Description: Substantive variation and multiple participant perspectives are presented within themes.

Location: Section 3
